# Assessing Predictive Ability of Dynamic Time Warping Functional Connectivity for ASD Classification

**DOI:** 10.1155/2023/8512461

**Published:** 2023-10-25

**Authors:** Christopher Liu, Juanjuan Fan, Barbara Bailey, Ralph-Axel Müller, Annika Linke

**Affiliations:** ^1^Department of Mathematics and Statistics, San Diego State University, California, USA; ^2^Brain Development Imaging Laboratory, Department of Psychology, San Diego State University, California, USA

## Abstract

Functional connectivity MRI (fcMRI) is a technique used to study the functional connectedness of distinct regions of the brain by measuring the temporal correlation between their blood oxygen level-dependent (BOLD) signals. fcMRI is typically measured with the Pearson correlation (PC), which assumes that there is no lag between time series. Dynamic time warping (DTW) is an alternative measure of similarity between time series that is robust to such time lags. We used PC fcMRI data and DTW fcMRI data as predictors in machine learning models for classifying autism spectrum disorder (ASD). When combined with dimension reduction techniques, such as principal component analysis, functional connectivity estimated with DTW showed greater predictive ability than functional connectivity estimated with PC. Our results suggest that DTW fcMRI can be a suitable alternative measure that may be characterizing fcMRI in a different, but complementary, way to PC fcMRI that is worth continued investigation. In studying different variants of cross validation (CV), our results suggest that, when it is necessary to tune model hyperparameters and assess model performance at the same time, a *K*-fold CV nested within leave-one-out CV may be a competitive contender in terms of performance and computational speed, especially when sample size is not large.

## 1. Introduction

Functional MRI is a noninvasive imaging technique that uses changes in blood oxygenation levels in the brain, called the blood oxygenation level-dependent (BOLD) signals, to detect regional brain activity. Brain regions that are activated during resting state or by cognitive tasks consume greater amounts of energy and oxygen due to metabolic activity. Following the activation, blood flow to the region is increased in order to replenish the region's supply of oxygenated blood. BOLD results from this outflow of deoxygenated hemoglobin and the inflow of oxygenated hemoglobin, and the phenomenon is detected by functional MRI due to the difference in magnetic properties between oxygenated and deoxygenated hemoglobin [1, 2]. The BOLD signals that are measured over the course of an MRI scan are used as an indirect measurement of localized brain functional activity [2], hence allowing for noninvasive study of brain functional activity patterns in the presence or absence of stimuli.

Functional connectivity MRI (fcMRI) is a specific application of functional MRI analysis that looks at the temporal associations between functional activity from distinct regions of the brain in order to study nondirectional connectivity patterns [2, 3]. The strength of temporal associations between the measured BOLD signals from distinct, functionally related regions of the brain has been the subject of research interest as a measure of “brain system integrity” [3], especially in how brain systems might differ between typically developing (TD) brains and those with developmental disorders. Interest in fcMRI research has steadily grown in part due to the applications of its findings towards developing a deeper understanding of how neurodevelopmental and neuropsychiatric disorders, such as autism spectrum disorder (ASD), affect the brain and its development. ASD is thought to be caused in part by disruptions of the normal range of functions that can be found in typically developing brains [4–6]. Although there are a number of other techniques, such as positron emission tomography (PET) and electroencephalography (EEG) that are used for neuroimaging research, functional MRI allows for noninvasive, high-resolution imaging of the brain, which makes it well poised for examining these potential differences. Beyond this, deeper understanding of brain system disruptions caused by developmental disorders can lead to more robust characterizations of the disorders themselves that may have significant clinical applications such as aiding in diagnosis through identification of disease biomarkers [3, 4]. Reliable diagnostic biomarkers could prove highly important in assisting with accurate clinical diagnosis of neurodevelopmental disorders that are primarily diagnosed through behavioral assessments and symptom observation that may leave room for subjectivity and misdiagnoses.

ASD is one such neurodevelopmental disorder that is diagnosed mainly through behavioral assessments. Current ASD diagnostic procedures can potentially be prone to subjectivity on the part of the clinician and may be affected by sociocultural factors [7], leading to either delayed diagnoses or misdiagnoses. In view of the generally accepted neurobiological nature of ASD, however, identification of reliable biomarkers is critical. Biomarkers can also provide a biological basis for further research into possible phenotypic subtypes of ASD [8, 9] and identify symptomatic differences that may exist among them.

Studies aimed at building highly accurate machine learning classification models of ASD with functional MRI data have been growing in popularity. Accurate classifiers that can reliably distinguish between typically developing participants and those with ASD can aid in efforts to identify potential imaging biomarkers of ASD by highlighting brain networks and functional connections that have the strongest discriminatory ability to differentiate the two groups. Some recent ASD classification studies that utilized machine learning methods with fcMRI data have achieved diagnostic classification accuracies above 70% [8, 10–13], suggesting that these data-driven models are able to capture diagnostically informative patterns and information. Although the results have been promising, biomarkers from individual classification studies have often failed to replicate in subsequent research.

### 1.1. Dynamic Time Warping as a Measure of Functional Connectivity

**Figure 1:**
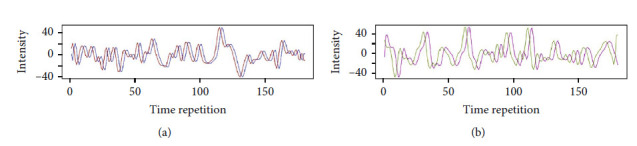
(a) Two plotted time series of a single BOLD signal; one plotted with a lag of 2 time points. The two time series have a PC of *r* = 0.49. (b) Two plotted time series of a single BOLD signal; one plotted with a lag of 3 time points. The two time series have a PC of *r* = 0.13.

Resting-state functional connectivity (FC) between distinct regions of the brain is traditionally measured with Pearson's correlation (PC) coefficient. The intuition behind resting-state FC is that distinct brain regions are deemed “functionally connected” if their respective BOLD signals show high levels of coactivation [3]. As the chosen measure for quantifying the level of coactivation, PC works well in instances where the compared signals are highly synchronous. However, if the compared signals exhibit time lags with regard to their activations or autocorrelation, the measured PC values are biased in ways that do not truly reflect the underlying similarity of the two signals. For example, Figure 1 depicts two instances in which measurements of PC between a signal and the same signal with a slight time lag can result in significantly lowered PC values. The potential for time lags and autocorrelations to result in low (or even negative) PC-estimated FC between otherwise highly similar time series is problematic. Hemodynamic responses, and by extension the measured BOLD signals, can vary across different regions of the brain with lags varying up to ±2 seconds within and between regions for reasons unrelated to neuronal activity [14]. These biological phenomena are potential cause for biases in the use of PC-measured FC between distinct resting-state brain networks that may obscure findings from functional connectivity studies.

Resting-state functional MRI studies of ASD have been subject to inconsistencies in results and general lack of replicability [15] due to methodological differences between studies such as data acquisition processes, data preprocessing pipelines, and the statistical models used for inference. These issues compound the potential drawbacks from using PC to measure FC in the presence of naturally occurring time lags and autocorrelated signals, motivating a need for alternative methodology with increased robustness in regard to these areas in order to increase the consistency of results from functional MRI ASD research.

Initially developed for speech recognition purposes [16], dynamic time warping (DTW) has been utilized in studies that analyze time series data for classification and clustering purposes [17, 18]. DTW has been proposed as a suitable alternative measure for functional connectivity between distinct brain regions that has been successfully employed in studies using electroencephalography (e.g., Karamzadeh et al. [19]), functional near-infrared spectroscopy (e.g., Eken [20]), and fMRI (e.g., Meszlényi et al. [21, 22]) to better understand clinical conditions such as fibromyalgia [23] and depression [24]. Unlike the Pearson correlations, DTW, when applied to fMRI time series, can take into account naturally occurring resting-state network BOLD signal time lags and autocorrelation as well as increased robustness to variations in preprocessing procedures with respect to global signal regression [21, 22, 25]. For these reasons, DTW is a potential candidate for use as an alternative measure of FC that is better suited to handle the aforementioned variations in hemodynamic responses and data preprocessing procedures and importantly potentially being able to improve consistency and replicability of functional MRI findings in ASD. Additionally, machine learning models trained on DTW-measured FC data have been found to yield greater prediction accuracies than those trained on PC-measured FC in classification studies utilizing resting-state fcMRI data [21, 22, 25], suggesting that DTW may be characterizing FC in a more informative way than PC for classification purposes. For these reasons, investigation into the efficacy of using DTW as the measure for FC may prove beneficial for our efforts to identify reliable brain imaging biomarkers of ASD.

### 1.2. Differences between Variants of Cross Validation

Many machine learning classification studies utilize fcMRI datasets with extremely high dimensionality in which the features greatly outnumber the sample size. For functional MRI ASD studies, recent developments and efforts such as the Autism Brain Imaging Data Exchange (ABIDE) [26] have been successful at increasing the number of large sample studies, but many studies still feature sample sizes ranging in the low hundreds while occasionally numbering in the tens [4] due to the additional complications and cohort effects that are introduced when using combined samples. When utilizing such low sample size in a machine learning study, the use of resampling methods to estimate model performance is necessary in place of traditional training-test set splits.

Cross validation (CV) is a technique that can be used for “honestly” estimating a model's performance, without the use of an external test set. In CV, a part of the dataset is “held out” while the remaining data is used to train the model. The trained model is then tested on the observations that were originally held out. These steps are repeated iteratively until all of the observations have been a part of the held-out set once. The model's performance is then estimated by its average performance over the various held-out sets. Many variants of CV, in addition to other resampling methods, have been applied to fcMRI machine learning studies as a way to utilize all of data to obtain unbiased estimates of model performance when working with low sample sizes [4, 11, 12, 27, 28]. The different variants of CV, such as *K*-fold CV and leave-one-out CV (LOOCV), function identically, but their model performance estimates differ due to the bias-variance trade-off. For example, LOOCV achieves approximately unbiased estimates, but at the cost of greater variance in comparison to *K*-fold CV for small values of *K* [29]. Beyond the bias-variance trade-off, some CV procedures can even introduce a bias into model performance estimates, hence producing optimistically biased results [30]. Given that these methods are heavily applied in fcMRI machine learning studies and that the prediction accuracy of the classifiers is one of the main measures used to assess how informative the features are, it is worth considering how directly comparable the results from different CV variants are and if the same inferences can be derived from the data and models regardless of which variant of CV was used. Understanding the extent of bias and variance associated with different implementations of CV is important in assessing the true predictive capabilities of the reported models and important features.

Our current study, based on the M.S. thesis work by one of the authors [31], seeks to investigate two main questions: (1) Does resting-state functional connectivity estimated with DTW have greater predictive ability for classifying participants with ASD in comparison to standard functional connectivity estimated by PC? (2) Do the different variants of CV produce similar model performance estimates and result in the same inferences? With the ultimate goal of identifying robust ASD imaging biomarkers, we use various machine learning methods to examine an alternative similarity measure for functional connectivity with the potential to be more informative for classification purposes, and we investigate the differences among the various CV procedures used to estimate model performance and assess how directly comparable these procedures are for inferences.

## 2. Data and Methods

In this section, we provide brief descriptions of our in-house data, dynamic time warping, the machine learning methods used including random forest and support vector machines, various methods of cross validation, and the design of our comparative study.

### 2.1. Data and Dynamic Time Warping

As our study builds upon previous work done by Linke et al. [25], we utilized the same dataset for our study. Each MRI scan was quality assessed through manual visual inspection by trained staff members. We selected a sample of participants that had high-quality scan data, while ensuring that our ASD and TD samples were matched for participant age, nonverbal IQ (NVIQ), and participant head movement during the MRI scans. Head movement was measured as the total root mean squared distance (RMSD) of each respective scan. This resulted in a final working sample of 99 participants (49 ASD). Summary statistics regarding the matching between our ASD and typically developing (TD) participants can be found in Table 1.

Our in-house data were collected on a GE 3T MR750 scanner with an 8-channel head coil at the UCSD Center for Functional MRI. Participants were instructed to keep their eyes open and fixated on cross-hair and not fall asleep over the duration of the scan. For each participant, functional T2^∗^-weighted images were obtained using a single-shot gradient-recalled, echo-planar pulse sequence that acquired 6-minute resting-state fMRI scans (180 time points with a repetition time = 2000 ms, echo time = 30 ms, slice thickness = 3.4 mm, flip angle = 90°, field of view = 22.0 mm, 64 by 64 matrix, and in-plane resolution = 3.4 mm^2^). High-resolution structural scans (180 slices, repetition time = 11.08 ms, echo time = 4.3 ms, 1 mm^3^ voxel dimension, flip = 8°, field of view = 256 mm, and matrix = 256 by 256) were also acquired for each participant from a FSPGR T1-weighted sequence. The preprocessing procedures for the acquired structural and functional MRI data are detailed in Linke et al. [25].

The FC estimates were derived from all regions of interest (ROI) defined in the Harvard-Oxford atlas [32], which is available in the CONN toolbox for MATLAB, resulting in 5460 unique FC measurements for each participant. The average time series were first computed from all voxels within each respective ROI, followed by calculations of the DTW and PC measurements between ROI pairs for each participant. DTW was calculated with MATLAB's DTW function, utilizing a warping window of 100 seconds (as shown to be optimal for fMRI time series data by Meszlényi et al. [21, 22]), and then converted into DTW similarity measures as in Meszlényi et al. [21, 22] and Linke et al. [25].

Dynamic time warping is a distance measure quantifying the dissimilarity between two time series, possibly of different lengths. Please refer to the Appendix for the details of its calculation. The typical distance measure for two vectors of the same length, say the Euclidean distance, is given by a summary of the point-wise distances. The DTW simply allows the signals to stretch or compress horizontally (along the time axis) and hence gives two time series that may exhibit time lags, but are similar otherwise, a small DTW dissimilarity (or high DTW similarity) value. Following recommendations by Meszlényi et al. [21, 22], we utilized a warping window size of 100 seconds in order to capture all potential BOLD signal lags. Once extracted, the DTW FC measurements were multiplied by -1 and then demeaned in order to turn them into DTW similarity measures as described in Meszlényi et al. [21, 22]. All references to DTW-measured FC in this paper refer to the DTW similarity measures.

**Figure 2:**
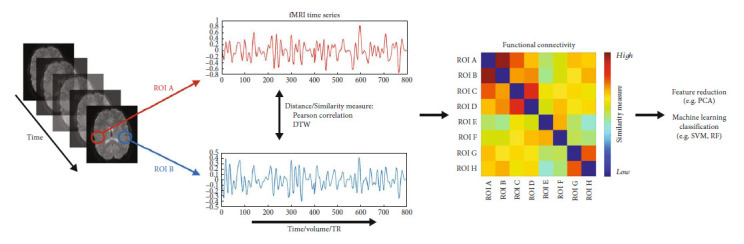
Time series are extracted from regions of interests (ROIs), and functional connectivity is calculated (using Pearson's correlation or DTW) between each ROI pair resulting in an ROI × ROI functional connectivity matrix for each participant. The matrices are triangulated, and pairwise ROI distance/similarity measures are used for machine learning classification.

The similarity estimates derived using dynamic time warping or Pearson's correlation between each ROI pair were used as features for the machine learning model. A basic diagram illustrating the methods is shown in Figure 2.

### 2.2. Machine Learning Methods

The machine learning models utilized in this study were support vector machines with the radial basis function kernel (SVM-Radial), support vector machines with the linear kernel (SVM-Linear), random forest (RF), and L_1_-regularized support vector machines (L_1_-SVM). The strong performance of SVM-Radial, SVM-Linear, and RF, as well as their relative ease of implementation and interpretation, has resulted in these three techniques being prominently featured in fcMRI research involving applications of machine learning methods. As such, they provide for a robust base for comparing the predictive abilities of FC estimated by the two measures. L_1_-SVM is a classifier that has built-in regularization that can eliminate superfluous functional connections from the data that are potentially uninformative or redundant [13, 33]. In case there is a significantly greater number of variables than available observations, as is generally the case with fcMRI data, machine learning methods can potentially be overfitted to small variations and patterns found within the dataset [13, 33] that do not improve the generalizability when applied to new data. Dimension reduction methods and regularized models alleviate some of these overfitting issues by either reducing the data into a lower-dimensional representation or outright eliminating variables. Beyond just combating overfitting, the use of regularization can be a data-driven way of exploring which functional connections are most informative for diagnostic classification of ASD.

#### 2.2.1. Random Forest

Random forest (RF) is an ensemble machine learning method that can perform both classification and regression, as well as handle high-dimensional data [34]. For classification, the RF model itself consists of a large number of separate decision trees that each provides their own predictions, and the class with the overall majority vote is the RF's resulting prediction. For each node of each decision tree within the RF, the respective best split comes from a random subset of all features, hence effectively “decorrelating” the trees by increasing the variance between them [34]. Although the decision trees that form the RF model are individually highly variable and weak in performance, the RF's use of an averaged prediction over all of the trees results in a highly accurate ensemble model.

Following recommendations by Breiman [34], we used square root of the number of features as the number variables to consider at each split (mtry). Additionally, the number of decision trees within each RF model was set to be 500, which was a sufficiently large number for ntree for the performance of our models to be stable. We decided not to treat mtry and ntree as hyperparameters for the following reasons: their values generally do not affect RF performance very much; there are well-established default values for mtry; for ntree, one can make sure that a large enough value is used so that the performance of RF is stable. With these two parameter sets, the RF had no hyperparameters that needed tuning when fitting the model. Our implementation of the random forest method was done with the randomForest package in R [35].

#### 2.2.2. Support Vector Machines

Support vector machine (SVM) is another classification model that has been widely used when studying the predictive performance of fcMRI data [4]. Given training data in vectors *x*_*i*_ ∈ *R*^*p*^, for *i* = 1, 2, ⋯, *n* (*n* observations with *p* features) and binary response vector *y*_*i*_ ∈ {−1, 1}, the general SVM algothrim solves for an optimal hyperplane, in possibly extended feature space, that best separates all the observations into two different classes. Three popular SVMs will be utilized in our study: SVM with the linear kernel which has a linear hyperplane as the decision boundary, SVM with the radial kernel which has a nonlinear decision boundary and emphasizes “local” points close to the observation whose class label is to be predicted, and an L_1_-regularized SVM which may be more suitable to the fcMRI data with high dimension and many noise variables. In this study, both SVM-Linear and SVM-Radial models were implemented using the kernlab package in R [36], while the L_1_-SVM model was implemented using the Liblinea R package [37].

All SVM models have a number of hyperparameters, for example, for controlling the number of observations that are allowed to be misclassified and the penalty for misclassifications. Allowing for some observations to be misclassified is necessary in instances where perfectly separating hyperplanes cannot be acquired and in order to avoid overfitting to the training data. We refer the reader to James et al. [29] for a general introduction to SVM models.

### 2.3. Cross Validation Methods

Resampling methods were necessary for our analysis as the total sample size was not large enough to justify a training and test set split for model building and performance assessment. *K*-fold CV is a standard technique used to estimate a model's true performance in which the full sample is split into *K* disjoint sets, called folds, and iteratively trained and tested on combinations of these *K* disjoint folds. For each iteration, one of the *K*-folds is held out and the model is trained on the remaining *K*-1 folds. The held-out set is then used to test the performance of the trained model. This process is repeated until each of the *K*-folds has been used once for model validation, and the average prediction accuracy over all *K* iterations is used as the estimate for the model's true accuracy. This procedure can also be used for tuning hyperparameters, in which one performs *K*-fold CV for various hyperparameter values of a model and then selects the set of hyperparameters that maximize the model's CV performance as the optimal model. When *K* is set to be equal to the sample size itself, so each held-out set is equal to a single observation, the procedure is referred to as leave-one-out cross validation, or LOOCV.

**Figure 3:**
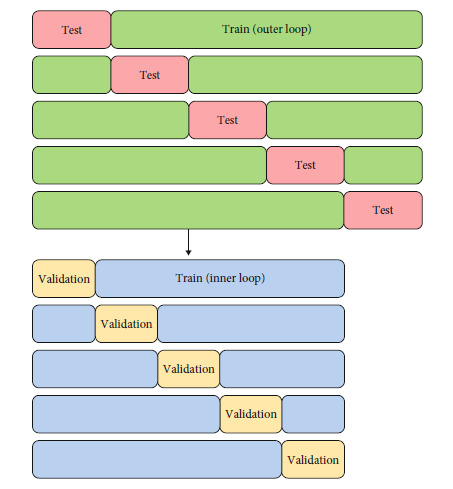
Visual demonstration of a nested CV algorithm with 5 outer folds and 5 inner folds. Notice that the fifth CV fold's training set (in green) is used to run an inner iteration of fivefold CV (the blue and yellow folds).

When hyperparameter tuning and performance assessment both need to be done, two iterations of CV must be performed in a “layered” fashion, called nested CV [30]. For each training and held-out set split in a regular CV procedure, nested CV performs a second iteration of CV on the training set. This second/inner iteration of CV is used for hyperparameter tuning in order to find an optimal model, and then, the optimal model is assessed on the original held-out set from first/outer iteration of CV. This double-layered process ensures that all of model fitting and performance assessment steps are done within the CV procedure and yields an “honest” estimate of the model's performance. An illustration of a nested CV procedure with both layers utilizing a 5-fold CV is given in Figure 3.

In our study, we also evaluate another type of CV procedure called the optimistically biased CV. This is essentially the same procedure introduced in Varma and Simon [30] where *K*-fold CV is performed for various sets of hyperparameter values for a model and the accuracy of the best performing set of hyperparameter values is used as the model's estimated performance. The estimated model performance is potentially biased (too optimistic) as hyperparameter tuning (part of the model building process) is not based on separate data from model assessment. The amount of bias from this specific procedure increases in situations when the signal-to-noise ratio is lower [38]. Nested CV was proposed by Varma and Simon [30] as an improvement on optimistically biased CV and is approximately unbiased [30]. However, nested CV is not always computationally feasible as performing nested iterations of CV can be very computationally expensive [38].

Feature processing and dimension reduction methods are possible to perform within the CV procedure, albeit precautions must be taken to implement them correctly. Incorrect implementation of these methods can lead to data “leaking” problems in which information from the testing sets is inadvertently used in the model building process. In our analysis, we also assessed our models' performances when the data were dimensionally reduced through principal component analysis (PCA). An incorrect implementation of PCA would be to use it to extract a number of principle components before performing CV with the dimensionally reduced data. This is due to all observations being used to extract the principle components, so information from testing set observations is used in the model building process. The correct implementation requires PCA to be performed for each training and held-out set split separately: for each iteration of CV, the principle components are extracted from the training set, and the held-out set is transformed according to these principle components. In this way, dimension reduction of the FC data can be done without any information from the testing sets “leaking” into the model building process and biasing the model performance estimates. All analyses were done using R [39].

### 2.4. Comparative Study

#### 2.4.1. Predictive Ability of fcMRI Data Measured with DTW versus PC

The primary goal of our comparative study is to compare the predictive ability for classifying ASD using functional connectivity estimated with DTW versus the standard approach of using functional connectivity estimated by PC. To this end, multiple machine learning models were fit on the two FC datasets separately, and the prediction accuracies from each model were used as measurement of the predictive capabilities of the respective measure. The datasets consisted of all 99 participants detailed in Section 2.1; the average time series was extracted for all cortical and subcortical ROIs given by the Harvard-Oxford atlas [32], and FC was measured with both DTW and PC for all ROI pairs. For the SVM-Linear, SVM-Radial, and L_1_-SVM models, 100 repeats of nested 5-fold CV (5-fold CV for both the inner and outer CV iterations) were performed with data based on either measure. This was repeated for all combinations of machine learning models, FC data measures, and whether PCA was performed to dimensionally reduce the data. Only the top 30 principle components from the data were used for model fitting. Model performance was estimated by the average prediction accuracy, sensitivity, and specificity over all 100 repetitions of nested 5-fold CV and used for comparison.

As our SVM-Linear, SVM-Radial, and L_1_-SVM models required hyperparameter tuning, we utilized nested CV procedures with these models. For hyperparameter tuning within each inner CV loop, we performed 80 iterations of random search instead of a grid search. Random search has been shown to be more efficient than other methods of hyperparameter tuning while being just as good, if not better, at producing near-optimal solutions [40] and hence was used exclusively to train the models. For RF, we set, a priori, parameter values for the number of trees and the number of variables to consider at each decision tree split, so there were no hyperparameters that required tuning. As a result, a regular *K*-fold CV procedure was sufficient for estimating the performance of our RF model. For RF, we performed 100 repetitions of 15-fold CV with and without the application of PCA to reduce our data to only the top 30 principle components.

#### 2.4.2. Assessing the Performance of Various CV Procedures

The secondary goal of our comparative study is to examine whether different variants of cross validation provide comparable results and similar inferences regarding our models' estimated ASD classification performance. To this end, two additional CV procedures were performed, combined with the SVM-Linear, SVM-Radial, and L_1_-SVM models, for comparison. The first procedure estimated model performance with a LOOCV. Within each loop of the LOOCV, each model's hyperparameters were tuned using a 3-fold CV with 40 iterations of random search; we call this procedure nested LOOCV. We specifically chose to use a nested CV procedure that combines LOOCV with 3-fold CV due to two important factors. The first is that this specific procedure was previously utilized by Plitt et al. [12], hence allowing our results to be directly comparable to previously published results. The second is due to its computational efficiency in comparison to our repeated nested 5-fold CV procedure. By reducing the number of random search iterations from 80 to 40 and performing only one iteration of nested LOOCV instead of 100 repeats of nested 5-fold CV, the necessary time for computation was reduced from hours to minutes. The second procedure was optimistically biased CV that was previously defined in Section 2.3. In particular, 10-fold CV was performed with 80 iterations of random search, and the highest resulting CV accuracy was chosen to be each model's estimated performance. We were interested in determining whether optimistically biased CV may give inflated model performance estimates compared to the other two CV procedures.

## 3. Results

**Figure 4:**
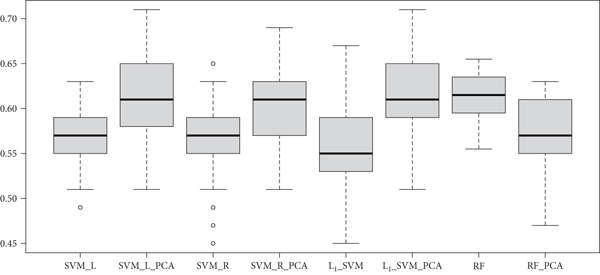
Box plots of CV-based accuracies comparing various models with the DTW-measured fcMRI data.

**Figure 5:**
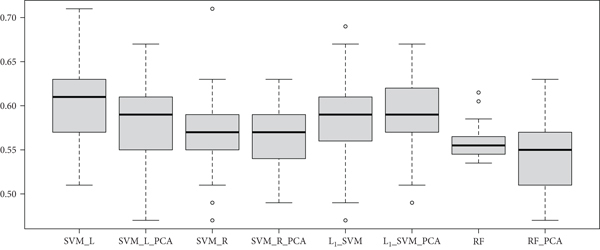
Box plots of CV-based accuracies comparing various models with the PC-measured fcMRI data.

**Figure 6:**
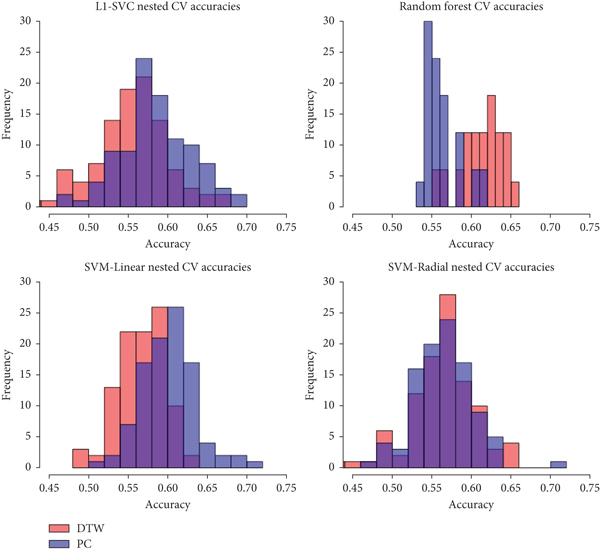
Comparison of ASD prediction accuracies over 100 iterations between DTW-measured versus PC-measured fcMRI data.

The mean accuracy, specificity, and sensitivity over 100 iterations of nested 5-fold CV for all models trained on DTW data can be found in Table 2; the corresponding results for all models trained on PC data can be found in Table 3. To compare the distributions of accuracy for all the models, box plots of the 100 accuracies for each model are presented in Figures 4 and 5 for DTW and PC-measured data, respectively. Note that the middle line in each box plot signifies the median accuracy. When the models were trained on the untransformed FC data, only RF reported a higher average estimated model performance with DTW data than with PC data. Histograms of the distributions of CV accuracies for all four models when trained on untransformed data are given in Figure 6. The distributions of CV accuracies between the two types of FC data overlap closely for SVM-Radial, suggesting that its performance was similar across the two types of data. For SVM-Linear and L_1_-SVM, the two models achieved greater average accuracy when trained on PC data than with DTW data, with peak CV accuracy reaching above 0.70 for SVM-Linear.

**Figure 7:**
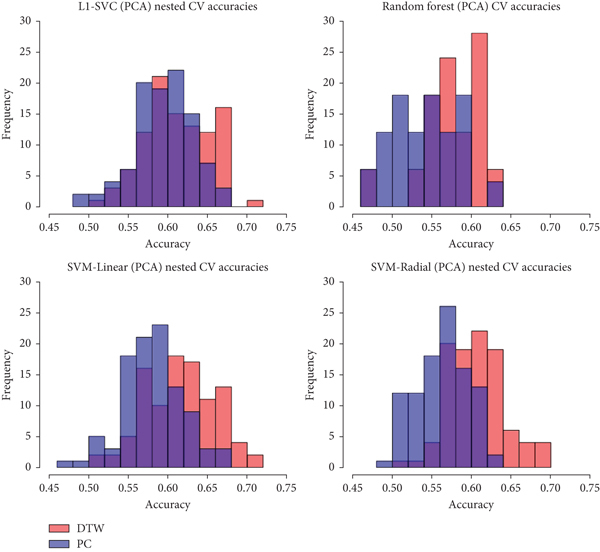
Comparison of ASD prediction accuracies over 100 iterations between the top 30 PCA components of DTW-measured versus PC-measured fcMRI data.

When PCA was utilized and the models were trained on only the top 30 principle components, the results indicate that FC measured with DTW is more predictive. All four models reported higher average CV accuracy when trained on PCA components extracted from the DTW-measured FC data, with SVM-Radial showing the largest difference in accuracy. Histograms of the distributions of all 100 iterations of CV for all four models when trained on the top 30 PCA components extracted from the FC data are given in Figure 7. The distributions of accuracies between the two types of FC data overlap closely for L_1_-SVM, while the remaining three models depict greater average accuracies for DTW-measured FC data, with peak CV accuracy reaching above 0.70 for SVM-Linear.

Dimension reduction through the use of PCA had mixed results with regard to classification accuracy. For the DTW data, the performance of L_1_-SVM, SVM-Radial, and SVM-Linear all improved when PCA was used. For PC data, however, the use of PCA had a very negligible effect on the performance of the same three models. In the case of RF, accuracy was higher for untransformed data regardless of the similarity measure used for FC, albeit the differences in accuracy were not large. This may be due to the fact that random forest can deal with high-dimensional features, so it can use the untransformed raw data effectively. Except for one case, the average sensitivity was higher than specificity for all models and both types of FC data; only L_1_-SVM trained on PCA dimensionally reduced PC-measured data reported higher specificity than sensitivity.

Results from the nested LOOCV and optimistically biased CV procedures are both given in Table 4. For nested LOOCV, the estimated model accuracies were in general very similar to the accuracies reported by the nested 5-fold CV procedure. The main differences were in the estimated model accuracies of the models trained on DTW data that utilized PCA for dimension reduction. For models trained on PC data, the accuracies show virtually no change when PCA is applied.

As expected, optimistically biased CV produced inflated model performances across all categories with the exception of SVM-Linear trained on PC data, in which its estimated accuracies were equal to the results of nested 5-fold CV. Similarly, optimistically biased CV yielded inflated model accuracies in comparison with nested LOOCV for all combinations of models and data except for SVM-Linear (PCA) trained on DTW data and SVM-Linear, with and without PCA, trained on PC data. The differences in CV accuracy between nested 5-fold CV and optimistically biased CV ranged from 0.02 to 0.11, with the largest difference being for SVM-Radial trained on DTW data.

## 4. Discussion

Results from our comparative study suggest that functional connectivity (FC) measured with DTW may be more informative for classifying ASD than FC measured with PC, especially when utilized with certain combinations of machine learning models and feature engineering methods. As we investigated model performances only with a single sample that came from data collected in house and preprocessed with a single preprocessing pipeline, it is still of interest to investigate if these results generalize to larger samples and different preprocessing pipelines.

Our results indicate that the additional regularization provided by the L_1_-SVM model did not improve model performance over the other nonregularized models. Although regularized and sparsity-inducing methods are an intuitive solution for working with high-dimensional data, the inability of regularization to yield better model performance in our case may be due to the type of regularization utilized. Regularization with the L_1_-norm has tendencies to indiscriminately eliminate all but one feature from any groups of correlated features as well as selecting to keep no more features than equal to the sample size when there are more features than observations [41]. This may be the cause of lower classification accuracies within our study as our sample size of 99 is considerably smaller than our 5460 measured FC per participant as well as the nature of FC itself. If there are more than 99 predictive connections, then information will be lost as the feature space will be constrained to a smaller number of connections than necessary. As for the tendency to indiscriminately eliminate all but one feature from groups of correlated features, this might prove to be an issue as fcMRI data might consist of correlated connections between functionally related brain systems. Although feature selection through regularization generally has a beneficial effect on model performance, regularization through the L_1_-norm might not be well suited to work with the inherent properties of high-dimensional resting-state fcMRI data.

Functional connectivity, with and without dimension reduction via PCA, was used as a feature in different models to predict ASD. With the exception of RF, dimension reduction through the application of PCA had mixed effects as it improved model performance only when used with DTW data. With that said, almost all models trained on DTW data showed notable improvements in CV accuracy when trained on only a subset of the principle components instead of the entire set of functional connections. This is consistent with Linke et al.'s [25] results as well, as their results of SVM-Linear models trained on DTW data having superior CV accuracies than PC data also utilized PCA for dimension reduction [25]. This may support the idea that DTW measures FC not in a strictly more informative way than PC but rather characterizes a different aspect of FC that may be complementary to PC and yield additional predictive information. Since RF can effectively deal with high-dimensional correlated features, dimension reduction via PCA is not necessary with RF and actually produces inferior results.

Our results suggest that the model performance estimates from nested LOOCV are in general the same as nested 5-fold CV, but with a larger effect on CV accuracy from dimension reduction. The large increase in accuracy is potentially due to the fact that our nested LOOCV procedure allows each model to be trained on more data than nested 5-fold CV. Since nested LOOCV uses held-out sets consisting of only 1 observation, the models are trained on 98 observations. In comparison, our nested 5-fold CV procedure uses held-out sets of 19 or 20 observations each, leaving the model to be trained with at most 80 observations at any given time. This difference in respective training set size may result in nested 5-fold CV producing slightly less fitted models on each CV iteration. The possible reduction in CV accuracies may be further compounded by being averaged over 100 repetitions as we did for the nested 5-fold CV procedure. Although the reported nested LOOCV accuracies of 0.71 and 0.67 for SVM-Linear and SVM-Radial are indeed higher than those from nested 5-fold CV, it is worth nothing that these values fall within the distribution of 100 repetitions of nested 5-fold CV, which can be viewed in Figure 7. These results suggest that nested LOOCV can function as an alternative to repeated nested *K*-fold CV that produces similar model performance estimates and insight into model behavior, but at a fraction of the computational cost.

In line with our expectations, our results saw inflated model performance when the optimistically biased CV procedure was performed in all but few instances. The largest increase in estimated model performance was 0.11 for SVM-Radial trained on the top 30 principle components extracted from DTW data. Although there seems to be an overestimation in CV accuracy from the optimistically biased CV procedure, further study would be necessary in order to assess the extent of the introduced bias, for example, a simulation study in which the true model performance is known.

The highest accuracy achieved in our study was 71%, which is higher than many previously reported high-quality studies using resting-state fMRI for classification of “autism.” Studies with higher accuracies may be overfitting or limiting their study participants to a more homogeneous small sample; see the review by Liu et al. [42]. Studies with limited sample size or participant heterogeneity are difficult to generalize to broader situations especially given the high heterogeneity in autism with respect to behavior, clinical symptoms, etiologies, cooccurring medical conditions, and other variables. Note that the purpose of the current study is not clinical diagnosis, i.e., classification of individual participants. Instead, the ultimate goals of our research are to improve our understanding of atypical brain network organization in autism and potentially identify subgroups in future research with larger samples and higher sensitivity.

One limitation of our study was that our selection of machine learning methods consisted mainly of classical machine learning models and dimension reduction methods that have been heavily used and studied in previous ASD classification studies. An extension of this study to include a wider range of methods, such as elastic net, which incorporates both L_1_ and L_2_ regularizations, or convolutional neural networks [21], may provide greater insight into the differences between the two measures for measuring FC. Additionally, DTW estimates of functional connectivity could further be optimized by tuning a DTW cost function rather than the absolute function [43] and by using recently proposed Amerced Dynamic Time Warping (ADTW) [44]. Similarly, prediction accuracy has been used as the performance measure in our study as it is the measure reported in many previous studies of ASD classification using resting-state fMRI. As pointed out by a reviewer, area under the receiver operating characteristic (ROC) curve may also be used as the yardstick for a future study.

## Figures and Tables

**Figure 8:**
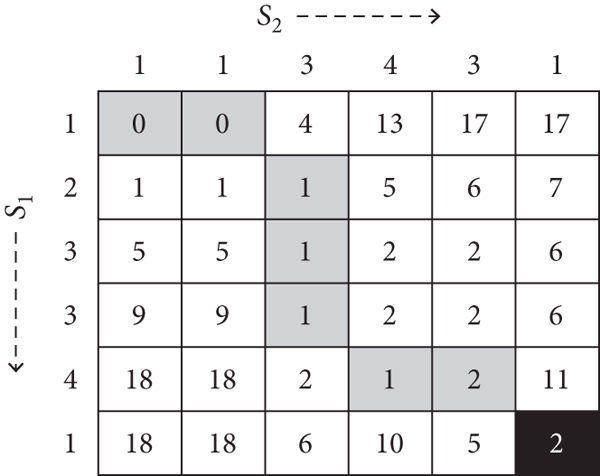
The calculated global cost matrix between two example time series, *s*_1_ = (1, 2, 3, 3, 4, 1) and *s*_2_ = (1, 1, 3, 4, 3, 1). The optimal matches between time points are highlighted in gray, and the resulting DTW distance between *s*_1_ and *s*_2_ is highlighted in black.

**Table 1:** Matching of our sample's participants across diagnostic groups, autism spectrum disorder (ASD), and typically developing (TD). The cell format follows mean ± SD [range]. SMD stands for standardized mean difference between the two groups. *p* values come from two-sample *t*-tests.

	ASD	TD	SMD	*p* value
*N*	49 (8 female)	50 (10 female)	—	—
Handedness	8 left	8 left	—	—

Age	13.65 ± 2.76 [7.4, 18]	13.32 ± 2.76 [8, 17.6]	0.12	0.55
RMSD	0.06 ± 0.03 [0.02, 0.11]	0.06 ± 0.03 [0.02, 0.14]	0.01	0.94
NVIQ	106.96 ± 19.36 [53, 145]	105.74 ± 13.36 [62, 137]	0.11	0.72

**Table 2:** Results from CV assessments of model performances with the DTW-measured fcMRI data.

Method (DTW)	Accuracy	Specificity	Sensitivity
Nested 5-fold CV			
SVM-Linear	0.57	0.52	0.62
SVM-Linear (PCA)	0.62	0.59	0.65
SVM-Radial	0.56	0.46	0.66
SVM-Radial (PCA)	0.61	0.59	0.63
L_1_-SVM	0.56	0.53	0.59
L_1_-SVM (PCA)	0.61	0.59	0.64
15-fold CV			
RF	0.61	0.56	0.66
RF (PCA)	0.58	0.48	0.67

**Table 3:** Results from CV assessments of model performances with the PC-measured fcMRI data.

Method (PC)	Accuracy	Specificity	Sensitivity
Nested 5-fold CV			
SVM-Linear	0.60	0.57	0.63
SVM-Linear (PCA)	0.58	0.57	0.59
SVM-Radial	0.57	0.52	0.61
SVM-Radial (PCA)	0.56	0.48	0.64
L_1_-SVM	0.59	0.55	0.62
L_1_-SVM (PCA)	0.60	0.61	0.59
15-fold CV			
RF	0.56	0.52	0.61
RF (PCA)	0.54	0.40	0.68

**Table 4:** Results from single iterations of nested LOOCV and optimistically biased CV.

Method	Accuracy	Sensitivity	Specificity
Nested LOOCV (DTW)			
SVM-Linear	0.58	0.51	0.64
SVM-Linear (PCA)	0.71	0.69	0.72
SVM-Radial	0.56	0.53	0.58
SVM-Radial (PCA)	0.67	0.67	0.66
L_1_-SVM	0.59	0.57	0.60
L_1_-SVM (PCA)	0.66	0.63	0.68
Nested LOOCV (PC)			
SVM-Linear	0.62	0.61	0.62
SVM-Linear (PCA)	0.62	0.61	0.62
SVM-Radial	0.54	0.51	0.56
SVM-Radial (PCA)	0.53	0.47	0.58
L_1_-SVM	0.59	0.53	0.64
L_1_-SVM (PCA)	0.59	0.61	0.56
Optimistically biased CV (DTW)			
SVM-Linear	0.60	0.57	0.62
SVM-Linear (PCA)	0.67	0.70	0.64
SVM-Radial	0.61	0.47	0.74
SVM-Radial (PCA)	0.67	0.61	0.72
L_1_-SVM	0.66	0.68	0.64
L_1_-SVM (PCA)	0.66	0.56	0.76
Optimistically biased CV (PC)			
SVM-Linear	0.60	0.54	0.66
SVM-Linear (PCA)	0.58	0.56	0.60
SVM-Radial	0.60	0.55	0.64
SVM-Radial (PCA)	0.63	0.48	0.78
L_1_-SVM	0.66	0.68	0.64
L_1_-SVM (PCA)	0.63	0.66	0.60

## Data Availability

All our data are uploaded to the National Institute of Mental Health Data Archive (NDA) https://nda.nih.gov.

## Calculation of the Dynamic Time Warping Distance

Let *s*_1_ and *s*_2_ denote two different time series of lengths *l*_1_ and *l*_2_, respectively. To calculate the DTW distance between *s*_1_ and *s*_2_, we begin by creating a *l*_1_ × *l*_2_ matrix, called the global cost matrix. Let the global cost matrix be denoted as *D* and let *D*(*i*, *j*) denote the (*i*, *j*)^th^ entry of *D*. *D*(*i*, *j*) corresponds to an accumulated warping distance between the prefix of length *i* from *s*_1_ and the prefix of length *j* from *s*_2_ and can be calculated as follows:

(A.1) $$\begin{array}{c} D\left(i,j\right)=\left\{\begin{array}{c} \left\|s_{1}\left(i\right),s_{2}\left(j\right)\right\|+\min\left\{D\left(i,j-1\right),D\left(i-1,j\right),D\left(i-1,j-1\right)\right\}\text{ if }i,j>1,\\ \left\|s_{1}\left(i\right),s_{2}\left(j\right)\right\|+D\left(i,j-1\right)\text{ if }i=1,j>1,\\ \left\|s_{1}\left(i\right),s_{2}\left(j\right)\right\|+D\left(i-1,j\right)\text{ if }j=1,i>1,\\ \left\|s_{1}\left(i\right),s_{2}\left(j\right)\right\|\text{ if }i=1,j=1. \end{array}\right. \end{array}$$

The DTW distance between *s*_1_ and *s*_2_ is given by the (*l*_1_, *l*_2_)^th^ entry of *D*, which we will denote as DTW(*si*, *sj*). Intuitively, the DTW distance is given by the path through *D* that minimizes the accumulated distance measures between the two time series. As noted in Equation (A.1), we used the Euclidean distance measure for calculating *D*, but any other distance measure can be substituted in place of it.

Figure 8 depicts the calculation of the global cost matrix and resulting DTW distance between *s*_1_ = (1, 2, 3, 3, 4, 1) and *s*_2_ = (1, 1, 3, 4, 3, 1). Beginning with the (1, 1)^st^ cell, each successive cell of the global cost matrix is calculated based on Equation (A.1). For example, the (4, 3)^th^ cell is given by

(A.2) $$\begin{array}{c} D\left(4,3\right)=\left\|s_{1}\left(4\right),s_{2}\left(3\right)\right\|+\min\left\{D\left(4,2\right),D\left(3,3\right),D\left(3,2\right)\right\}={\left(3-3\right)}^{2}+\min\left(1,5,9\right)=1. \end{array}$$

The rest of the global cost matrix is filled out sequentially until the very last cell. The resulting DTW distance between *s*_1_ and *s*_2_ is the (*l*_1_, *l*_2_)^th^ = (6, 6)^th^ cell of the global cost matrix, which is highlighted in black. Each cell within the global cost matrix can be thought of as the cost associated with matching the time points corresponding to that row and column; the more dissimilar they are, the greater the cost of that matching. Since each step of the DTW algorithm adds the minimal cost from the preceding matches, we can think of the resulting DTW distance between *s*_1_ and *s*_2_ as the optimal transformation that warps one time series to be as similar as possible to the other.

Note that the DTW algorithm allows for one-to-many matchings between time points in order to handle intermittent phase delays between the two time series (spontaneous compressions or expansions with respect to time). This one-to-many matching is visible in the global cost matrix as any series of vertical or horizontal optimal matches (gray highlighted cells).
